# An Unusual Retinal Vessel Modification in Patients Affected by JIA-Uveitis with a Follow-Up Longer Than 16 Years

**DOI:** 10.1155/2020/4720819

**Published:** 2020-01-28

**Authors:** Alessandro Abbouda, Irene Abicca, Simone Bruschi, Federico Ricci, Gianluca Aloe, Maria Pia Paroli

**Affiliations:** ^1^Department of Ophthalmology, Sapienza University, Umberto I Hospital, Rome, Italy; ^2^IRCCS-Fondazione Bietti, Rome, Italy; ^3^UOSD Retinal Pathology PTV Foundation Policlinico Tor Vergata University, Rome, Italy

## Abstract

**Purpose:**

To report unusual and rare clinical changes of retinal vessel pattern in a series of patients affected by Juvenile Idiopathic Arthritis (JIA) uveitis with a follow-up longer than 16 years.

**Methods:**

A series of three patients with JIA-uveitis followed at the University of Rome “Sapienza” from 1998 to 2014 were reported. The retinal vessels were analyzed with fluorescein angiography using Heidelberg Retinal Angiogram-2 (HRA-2; Heidelberg Engineering GmBH, Dossenheim, Germany) and the Topcon TRC-50LX retinal camera (Topcon Europe, The Netherlands). A Spectralis Domain OCT (SD-OCT) (Spectralis Family Heidelberg, Germany) was performed to evaluate vessel anatomy.

**Results:**

Fundus photography showed sheathed vessels localized around the optic disc in every case. Angiography revealed a normal physiology of vessel walls and flow; no sheathing or leakage of dye was observed. SD-OCT demonstrated reflective vessel walls. Vessel lumen appeared patent, and the normal “*hourglass configuration*” was blurred, but identifiable.

**Conclusions:**

Vessel modifications observed in long-standing JIA-uveitis are not signs of vascular inflammation and are not associated to hypoperfusion. In these cases, ophthalmologists should avoid further invasive investigation and should consider introducing SD-OCT as a routine method to evaluate the vessel changes during the follow-up.

## 1. Introduction

Juvenile Idiopathic Arthritis (JIA) is the most common systemic disease associated with uveitis in childhood. A significant number of patients have already been affected by ocular complications at the time of the diagnosis of uveitis. The ocular complications and visual loss in these patients were investigated in several studies [1–10].

We reported a clinical series of three patients affected by long-term JIA-uveitis with a follow-up of 16 years who developed unusual retinal vessel pattern modifications localized around the optic disc.

## 2. Materials and Method

This study evaluated three patients with diagnosis of JIA-uveitis followed at the University of Rome “Sapienza” from 1998 to 2014. The patients were classified according to the criteria of the International League against Rheumatism (ILAR) [11] and with the International Uveitis Study Group recommendations (IUSG) [12].

Retinal vessels were analyzed with fluorescein angiography using Heidelberg Retinal Angiogram-2 (HRA-2; Heidelberg Engineering GmBH, Dossenheim, Germany) for two patients (cases 1 and 2) and the Topcon TRC-50LX retinal camera (Topcon Europe, The Netherlands) in one patient (case 3). A Spectralis Domain OCT (SD-OCT) (Spectralis Family Acquisition Module, V 5.1.6.0; Heidelberg Engineering, Heidelberg, Germany) was performed to evaluate vessel anatomy [13].

## 3. Case Presentation

**Figure 1:**
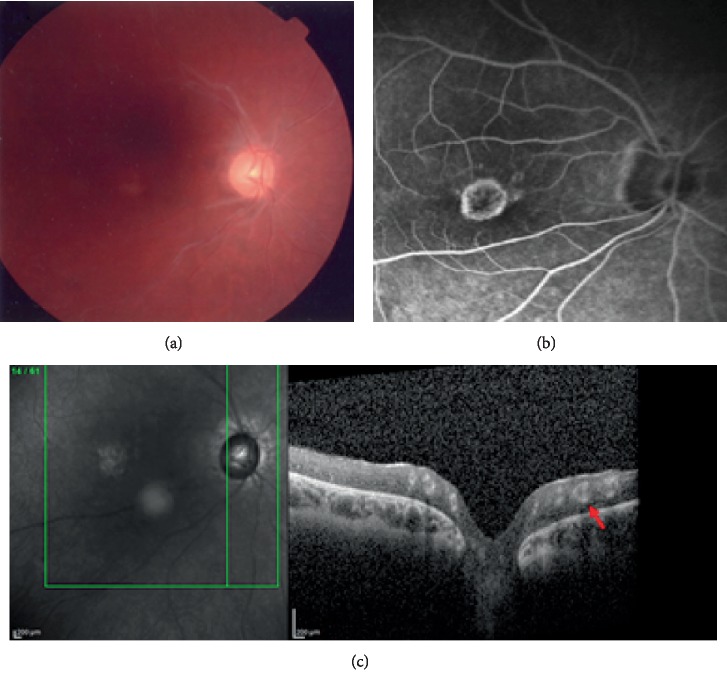
Case 1. (a) Fundus photography confirmed visible sheathed vessels. (b) FA; posterior pole showed a normal appearance of vessel walls and flow; no sheathing or leakage of dye was observed at any time. Window effect appears in the foveal area due to atrophy of retinal pigment epithelium subsequent to chronic macular edema. (c) SD-OCT line scan showed very reflective vessel wall. The hyperreflection involves the entire vessel walls, and there is no difference in reflectivity between veins and arteries. Vessel lumen appears patent, and the internal hourglass configuration is blurred, but identifiable.

Case 1 is a 29-year-old woman with bilateral uveitis onset at 5 years old associated with pauciarticular JIA diagnosed one year before. She referred to our centre at the age of six with bilateral cataract, band keratopathy, and glaucoma. Optic disc edema was detected by ocular B-scan echography. Anti-nuclear antibody (ANA) was positive (1 : 80) and rheumatoid factor (RF) negative. Haplotype HLA-B27 and DR11 antigens were absent. At the age of ten, phacoemulsification was performed in the right eye. During the follow-up, she developed several episodes of macular edema. To control eye inflammation, topical oral and peribulbar steroids were administered. Glaucoma was managed by topical drops. At 25 years old, right fundus examination underlined a sheathing-like aspect of vessels at the emergence of the optic disc. The left eye was not evaluable due to media opacities. Fundus photography, FA, and SD-OCT were performed (Figure 1).

**Figure 2:**
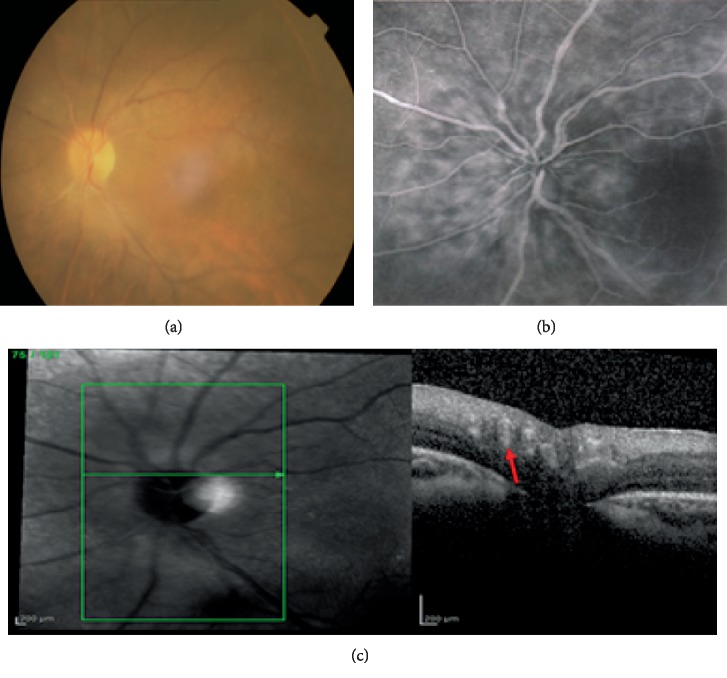
Case 2. (a) Fundus photography described sheathed vessels emerging from the optic disc. (b) FA showed normal vessel walls and flow. (c) The vessel wall reflectivity seemed to be normal or slightly hyperreflective in the SD-OCT linear scan (red arrow).

Case 2 is a 21-year-old woman affected by bilateral JIA- uveitis associated to oligoarthritis since the age of six. She presented at our centre with bilateral cataracts, band keratopathy, and seclusio pupillae. ANA were positive (1 : 40) and RF negative. The haplotype HLA-B27 and DR11 antigens were positive. At the age of eight, she underwent cataract extraction by pars plana lensectomy with anterior vitrectomy in both eyes. At nine years old, she developed optic disc edema in both eyes with three months of interval between each one. At the age of eleven, macular edema was diagnosed in the left eye. She was treated with oral, topical, and periocular corticosteroids. When she was 21, fundus evaluation showed sheathed vessels emerging from the optic disc in both eyes (Figure 2).

**Figure 3:**
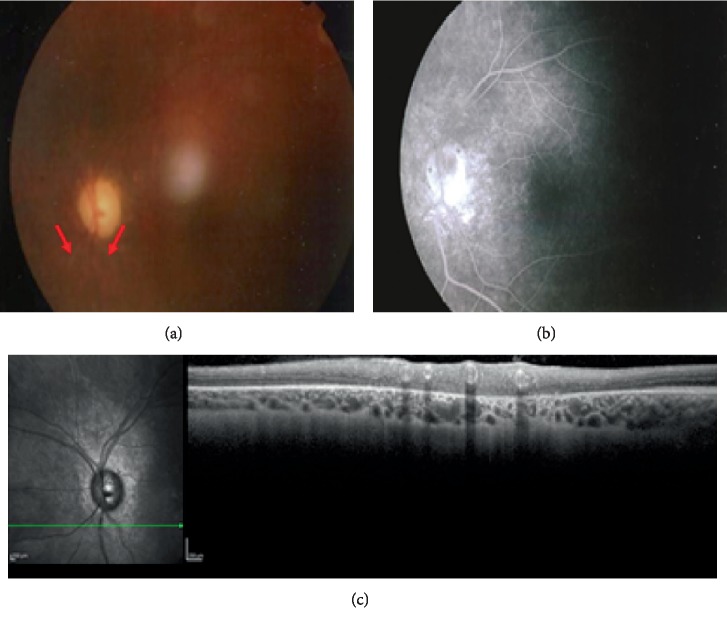
Case 3. (a) Sheathed aspect of vessels emerged from the lower bound of the optic nerve head (red arrows), blurred image due to optical opacities. (b) Late stages of the angiogram demonstrate normal fluorescence of the vascular tree. Staining was noted on the inferotemporal border of the optic nerve related to chorioretinal atrophy. (c) SD-OCT scan demonstrated typical internal hourglass configuration and a highly reflective vessel wall in both arterioles and veins.

Case 3 is a 20-year-old woman, diagnosed of bilateral JIA-uveitis since the age of five. Articular involvement occurred when the patient was 11 years old. At the first consultation, she had band keratopathy, seclusio pupillae, cataract, and glaucoma in both eyes. Bilateral optic disc edema was found one month after the onset, and it was confirmed using B-scan echography. HLA-B27 was negative and HLA-DR11 positive. ANA were positive (1 : 320). Lensectomy with pars plana vitrectomy was performed in both eyes at 7 and 8 years of age. At 10 years old, she underwent trabeculectomy in the left eye. Topical and oral corticosteroids were not enough to control eye inflammation. At 11 years old, oral cyclosporine was started then replaced with methotrexate. It was associated to infliximab two years later. At 16 years old, adalimumab was introduced for articular recrudescence. She was followed up regularly, and when she was 20 years old, sheathed vessels around the optic disc appeared in both eyes (Figure 3).

They all underwent screening tests for systemic vasculitis including antineutrophil cytoplasmic antibodies, anticardiolipin antibody, lupus anticoagulant, anti-DNA antibody, and anti-Ro/SSA and La/SSB antibodies. Screening results were negative in all three cases.

## 4. Discussion

Vasculitic process is not a common feature of this disease, and the differential diagnosis between vasculitis and other vascular modifications is fundamental. FA in active vasculitis includes vascular staining and leakage of dye due to the breakdown of the inner blood-retinal barrier with typical “skip lesions,” capillary nonperfusion, retinal neovascularization, and sclerosis of vessels. In our cases, FA evidenced no signs of vascular leakage in both arteries and veins. The process is confined in the area around the optic nerve disc and differs from vasculitis where it is normally located in the middle and peripheral retina. No signs of intraretinal haemorrhages, cotton wool spots, or vascular occlusion were detected.

Normal vessels appear using SD-OCT, with an oval- or round-shaped form and a heterogeneous reflectivity. The top and bottom of the vessel walls, which are vertical to the SD-OCT light source, show the innermost and outermost hyperreflectivity. The interior of vessels shows hourglass-shaped or a double “c” pattern and is due to physiologic blood flow [13, 14]. In our cases, the typical internal hourglass configuration is preserved, indicating that lumen is patent, but consisting diffuse higher reflectivity of the walls was found compared to normal vessels.

Iwasaki et al. [15] described a marked increase and disarrangement of collagen fibrils in the media and adventitia in patients with various vascular diseases. These alterations were associated to the changes on smooth muscle cells and their laminae causing a modification of the transparency of the vessel walls. The lumens of most sheathed vessels were still patent, and the blood cells and endothelial cells appeared to be normal. Regarding our cases, we thought that bilateral optic disc edema during the childhood may induce a progressive collagen fibril disarrangement and confer a sheathed-like aspect to the peripapillary vessels.

Although SD-OCT was a promising additional tool for the assessment of vessel anatomy, it currently can be replaced by swept-source wide-field optical coherence tomography angiography (OCTA). OCTA has the advantage to visualize the blood flow in various layers of the retina without having to inject the dye [16], and recently, capillary nonperfusion in intermediate uveitis has been detected [17]. Unfortunately, when these three patients were followed, OCTA was not available and SD-OCT was the only noninvasive valuable tool for the analysis of sheathed vessels.

SD-OCT optic disc vessel abnormalities in JIA-uveitis patients have not previously been reported in the literature. However, widening of the small peripapillary veins and a significantly larger number of veins without branching were reported in patients with bilateral optic disc edema associated to idiopathic intracranial hypertension and cavernous sinus thrombosis [18].

Overall, based on our cases, we can conclude that SD-OCT could be introduced as a routine study method to evaluate vessel wall reflectivity for the long-term follow-up of JIA- uveitis before performing further investigations.
